# cellsig plug-in enhances CIBERSORTx signature selection for multidataset transcriptomes with sparse multilevel modelling

**DOI:** 10.1093/bioinformatics/btad685

**Published:** 2023-11-11

**Authors:** Md Abdullah Al Kamran Khan, Jian Wu, Yuhan Sun, Alexander D Barrow, Anthony T Papenfuss, Stefano Mangiola

**Affiliations:** Department of Microbiology and Immunology, The University of Melbourne at The Peter Doherty Institute for Infection and Immunity, Parkville, VIC 3010, Australia; Cancer Biology And Therapy, Olivia Newton-John Cancer Research Institute, Heidelberg, VIC 3038, Australia; Department of Microbiology and Immunology, The University of Melbourne at The Peter Doherty Institute for Infection and Immunity, Parkville, VIC 3010, Australia; Department of Microbiology and Immunology, The University of Melbourne at The Peter Doherty Institute for Infection and Immunity, Parkville, VIC 3010, Australia; Division of Bioinformatics, The Walter and Eliza Hall Institute of Medical Research, Parkville, VIC 3010, Australia; Department of Medical Biology, University of Melbourne, Melbourne, VIC 3010, Australia; Division of Bioinformatics, The Walter and Eliza Hall Institute of Medical Research, Parkville, VIC 3010, Australia; Department of Medical Biology, University of Melbourne, Melbourne, VIC 3010, Australia

## Abstract

**Motivation:**

The precise characterization of cell-type transcriptomes is pivotal to understanding cellular lineages, deconvolution of bulk transcriptomes, and clinical applications. Single-cell RNA sequencing resources like the Human Cell Atlas have revolutionised cell-type profiling. However, challenges persist due to data heterogeneity and discrepancies across different studies. One limitation of prevailing tools such as CIBERSORTx is their inability to address hierarchical data structures and handle nonoverlapping gene sets across samples, relying on filtering or imputation.

**Results:**

Here, we present cellsig, a Bayesian sparse multilevel model designed to improve signature estimation by adjusting data for multilevel effects and modelling for gene-set sparsity. Our model is tailored to large-scale, heterogeneous pseudobulk and bulk RNA sequencing data collections with nonoverlapping gene sets. We tested the performances of cellsig on a novel curated Human Bulk Cell-type Catalogue, which harmonizes 1435 samples across 58 datasets. We show that cellsig significantly enhances cell-type marker gene ranking performance. This approach is valuable for cell-type signature selection, with implications for marker gene validation, single-cell annotation, and deconvolution benchmarks.

**Availability and implementation:**

Codes and the interactive app are available at https://github.com/stemangiola/cellsig; and the database is available at https://doi.org/10.5281/zenodo.7582421.

## 1 Introduction

The estimation of cell-type representative transcriptomes is an essential endeavour in modern biology, facilitating the molecular characterization of cellular lineages, enabling precise single-cell annotation (Aran *et al.* 2019) aiding in the deconvolution of bulk transcriptomes (Newman *et al.* 2015, Monaco *et al.* 2019), and serving as valuable clinical predictors (Foroutan *et al.* 2021, Pal *et al.* 2021, Sun *et al.* 2021). Single-cell RNA sequencing resources like the Human Cell Atlas (Osumi-Sutherland *et al.* 2021) have become invaluable for cell-type profiling. To scale cell-type signature estimation to vast single-cell datasets with thousands of biological samples, a common practice is aggregating single cells into pseudobulk samples. This approach reduces data dimensionality and enables the utilization of established analytical tools (Squair *et al.* 2021). In parallel, cell-type-specific bulk transcriptomes obtained through experimental purification methods, such as antibody-based flow-sorting, represent a rich and independent resource, as cell types are discerned based on protein expression rather than RNA abundance. While the exponential growth in the availability of cell-type transcriptional profiles promises unprecedented opportunities, it is accompanied by significant challenges. These challenges arise from the heterogeneity across data sources. Samples within each study tend to exhibit self-similarity based on the experimental design, and some datasets possess an order of magnitude more samples than others, potentially skewing the transcriptional representation of cell types. In addition, gene sets may partially overlap across datasets.

CIBERSORTx (Newman *et al.* 2019), a widely adopted tool for estimating cell-type transcriptional profiles from cell-type-specific transcriptomes, relies on complete gene overlap among samples and does not account for the hierarchical structure of the data. Adjusting for multilevel effects (e.g. study) and handling nonoverlapping gene sets could address these limitations and enhance the signature estimation performance of this popular method. Multilevel (i.e. random-effect) statistics can model hierarchical sources of variability, including samples and studies, which can avoid weighting biases when distinct studies have wildly different sizes. Here, we introduce cellsig, a novel plugin method designed to augment the signature estimation capabilities of CIBERSORTx by adjusting transcriptomes for hierarchical effects (study/dataset) and addressing data sparsity.

cellsig is a Bayesian multilevel generalized linear model tailored to RNA sequencing data. It uses joint hierarchical modelling to preserve the uncertainty of the mean-variability association of the gene-transcript abundance [similar to the outlier detection method ppcseq (Mangiola *et al.* 2021b)]. The joint modelling of mean-variability association, performed on latent parameters rather than observed data, does not require harsh filtering of lowly abundant gene transcripts, which is key for modelling cell-type-specific markers transcribed in specific cell populations. Also, cellsig models nonoverlapping gene sets across samples as missing information.

Although multilevel models already exist (Bates *et al.* 2015, Yirga *et al.* 2020), they do not actively allow data adjustment. Those methods and other nonmultilevel that are tailored to RNA sequencing, such as edgeR (Robinson *et al.* 2010), DESeq2 (Love *et al.* 2014), and limma-voom (Law *et al.* 2014) estimate feature-wise dispersion from the mean-variability association; however, the uncertainty of this association is not part of the model, and requires filtering of the lowly abundant genes (Mangiola *et al.* 2021a,b). Also, the current methods do not model missing information but rely on external imputation.

To evaluate the effectiveness of cellsig, we tested the performance in deconvolution and gene ranking of CIBERSORTx-generated signatures with or without multilevel adjustment. We tested cellsig on the novel Human Bulk Cell-type Catalogue (HBCC), a comprehensive resource harmonizing 1435 samples from 58 diverse datasets, encompassing 67 distinct cell types. Accessible via a user-friendly web interface (https://github.com/stemangiola/cellsig), this catalogue is an invaluable resource for bulk and single-cell-based research, enabling specific biological investigations through its intuitive interface. We show that cellsig improves cell-type marker gene ranking performance, resulting in higher deconvolution accuracy.

In conjunction with the HBCC, our method represents a valuable resource for cell-type signature selection, with profound implications for marker gene validation, single-cell annotation, and deconvolution benchmarks. cellsig empowers researchers to estimate cell-type signatures from large-scale complex cell-type transcriptional catalogues, further advancing our understanding of cellular diversity and function.

## 2 Materials and methods

### 2.1 Data acquisition and harmonization

We collected 58 transcriptome datasets (Table 1), including experimentally purified (e.g. cell sorting or bead purification) or primary culture (only controls) samples. Most samples were derived from tissue; few were derived from *in vitro* activation assays (including cases of type-I and II macrophages and conventional dendritic cells). We procured the raw read counts for most datasets (*n* = 53) with an exception for a few (*n* = 5), which only contained the normalized read counts. We processed and aligned the RNA-seq reads from the raw paired-end FASTQ files for some of the datasets that do not have the read counts information available. Firstly, we trimmed the sequencing adapter sequences by the Trim-Galore tool (Krueger 2015) running on a paired-end option with other parameters set to default. The quality of the trimmed reads was checked through the FastQC tool (Andrews 2010). Finally, the read alignment was achieved through the STAR aligner (Dobin *et al.* 2013) against the human genome annotation data from assembly version GRCh38, and the default alignment parameters were utilized.

**Table 1.** Datasets included in the HBCC database.

Name	Samples	Cell types	Reference
ENCODE	382	25	Luo *et al.* (2020)
BLUEPRINT	84	29	Fernández *et al.* (2016)
GSE107011	106	19	Monaco *et al.* (2019)
Other sources	770	117	*(see Supplementary Table S4)*

After the acquisition, datasets were further processed to have consensus gene feature keys across the datasets; for instance, NCBI gene IDs/Ensembl gene IDs were converted to the associated HGNC gene symbols using the ‘AnnotationDbi’ and ‘org.Hs.eg.db’ packages. In cases of annotating multiple IDs to the same gene symbol, we only selected the information of the first entry. We also dropped the transcripts which were not annotated to any gene symbols from the gene-ID conversion process. Next, duplicated transcript-abundance pairs were aggregated to have a unique transcript-abundance value for each gene across every sample. In addition, we filtered out the transcripts with missing abundance counts. Finally, all the datasets were uniformly structured to have the following information: gene symbol, sample IDs, cell-type nomenclature from the source dataset, and reference ID of the source dataset.

As the collected datasets were assembled from several studies with diversified experimental designs, they contained samples with varying cell-type nomenclatures despite their biological and functional similarities. Therefore, to harmonize the samples representing a well-defined broad cell-type group, we redefined the source-cell-type—nomenclatures of the samples with similar cellular characteristics to a more conventional cell-type nomenclature (Supplementary Table S1). For instance, we specified the cell type as ‘CD56^dim^ NK’ for the samples we phenotypically characterized as CD56^dim^CD57^+^, CD56dimCD57^−^, CD56^dim^CD94^+^, CD56^dim^CD94^−^, and CD56d^im^CD16^+^ NK cells.

To remove potentially duplicated samples from the catalogue, we filtered samples from the same dataset through the tidybulk package (remove_redundancy function) (Mangiola *et al.* 2021a). The top 1000 most-variable genes of each sample within a dataset are screened first, and then the pairwise Pearson correlation coefficients were estimated between all the samples of that dataset. Finally, samples with a correlation value >0.99 were dropped from the catalogue. Following this workflow, from the initially gathered 1629 samples, we ended up with 1435 nonredundant transcriptome samples, which constructed the final catalogue (Supplementary Table S2).

### 2.2 Bayesian sparse random-intercept model of transcript abundance

**Figure 1.**
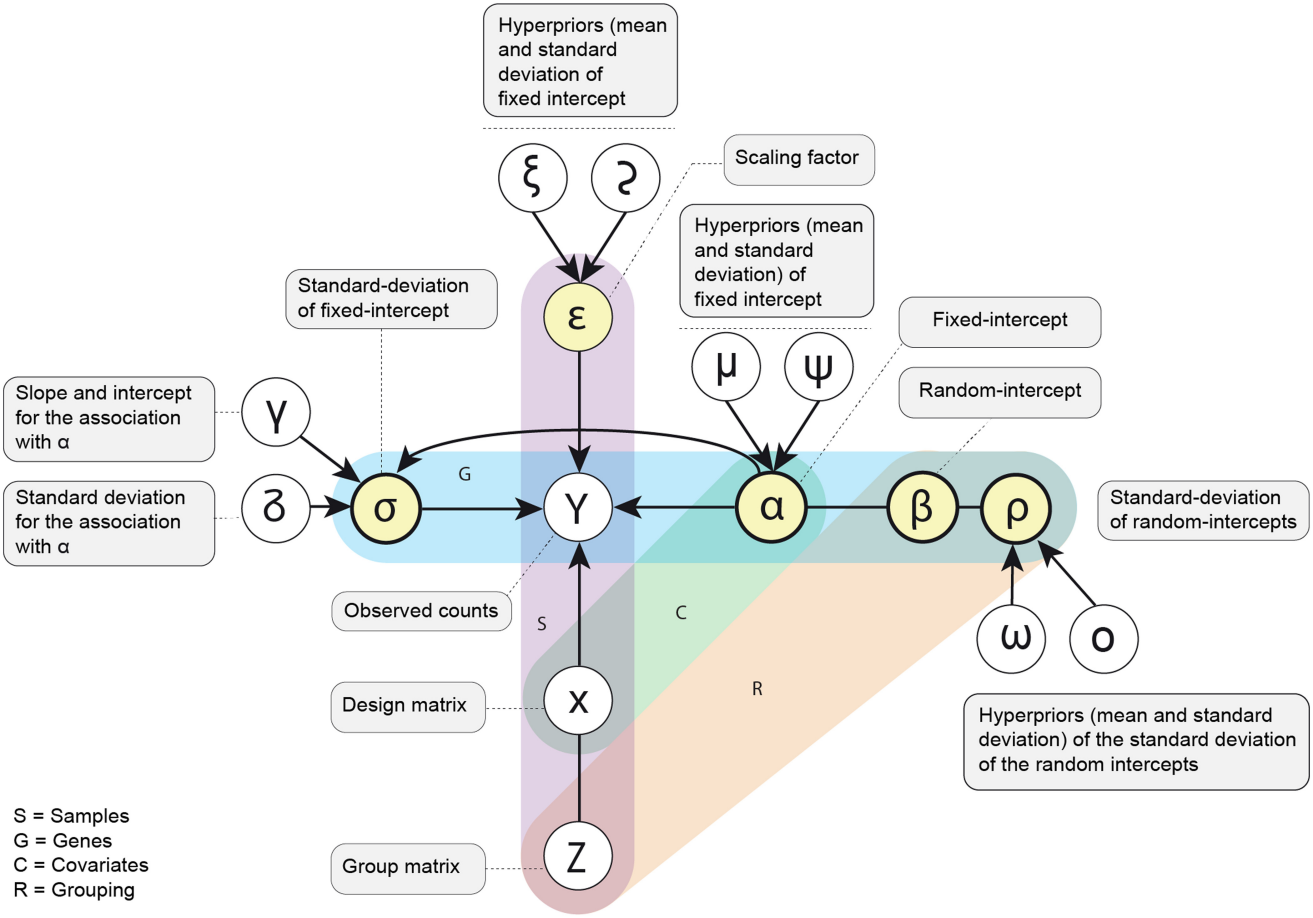
Graphical plated representation of the inference model representing the Equations (1–15). The empty circles represent observed data or the constants of the hyperpriors given as data. The full circles represent parameters (reals, vectors, or matrices). The shaded frames represent the dimensionality of the variables. The squares include the description of the variables.

**Noise model:** The regression model (Figure 1) is based on a negative binomial distribution. The binomial distribution is a discrete probability distribution with a lower bound at zero. This distribution models the number of failures in a series of independent and identically distributed Bernoulli trials up to a specified number of successes. The negative binomial is here parameterized by mean (log link) and overdispersion (log link) [Equation (1)]. This parameterization is convenient for our linear modelling.


(1) $$NB\;\left(n|\mu ,\;\theta \right)=\left(\begin{array}{c} n+\;\theta -1\\ n \end{array}\right)\;{\left(\frac{\mu }{\mu +\;\theta }\right)}^{n}{\left(\frac{\theta }{\mu +\;\theta }\right)}^{\theta }$$

**Mean:** The mean transcript abundance (log link) is modelled as a combination of a group-level [i.e. random; e.g. dataset; Equation (2)] effect and a population-level (i.e. fixed) effect. The mean of the negative binomial distribution for each gene is calculated as the sum of the expected value for the population and the group-level transcript abundance.


(2) Xα+Zβ

**Group-level mean and standard deviation:** The variability across group effects is modelled gene-wise with the standard deviation parameter *ρ* [Equation (3)]. *ω* and o are the mean and standard deviation hyperpriors of *ρ* [Equations (4) and (5)].


(3) $$\beta \;∼\;\mathrm{normal}\left(0,\;\rho \right)\;$$

(4) $$\rho \;∼\;\mathrm{gamma}\left(\omega ,\;o\right);\mathrm{with}\;\omega >1\;\mathrm{and}\;o>1$$

(5) $$\omega ,o\;∼\;\mathrm{normal}\left(3,\;1\right)$$

**Population-level overdispersion:** The overdispersion is modelled gene-wise by the parameter *σ*. The parameter *σ* represents the negative log overdispersion from Equation (10). The prior of the overdispersion parameter *σ* is conditional to the means *α* [Equation (6)]. The positive association between the log mean and its log over-dispersion is modelled as a hierarchical linear function. γ0 and γ1 are the intercept and slope of the log overdispersion-log mean association. δ is the standard deviation. These parameters have hyperpriors defined by variables set outside the model, informed by the datasets analyzed in this study and left nonstringent, and can alternatively be set by the user [Equations (7–9)]. Given that those hyperpriors are externally provided, we identified that setting the standard deviation at 20% of the mean achieved model convergence while allowing flexibility.


(6) $$\sigma_{g}\;∼\;\mathrm{normal}\left(\lambda_{0}+\lambda_{1}*\;\alpha_{g},\delta \right)$$

(7) $$\lambda_{0}\;∼\;\mathrm{normal}\left(\lambda_{0}^{*},\;\frac{{|\lambda }_{0}^{*}|\;}{5}\right);\;\mathrm{where}\;\lambda_{0}^{*}\;\mathrm{is}\;\mathrm{calculated}\;\mathrm{outside}\;\mathrm{the}\;\mathrm{model}$$

(8) $$\lambda_{1}\;∼\;\mathrm{normal}\left(\lambda_{1}^{*},\;\frac{{|\lambda }_{1}^{*}|\;}{5}\right);\;\mathrm{where}\;\lambda_{1}^{*}\;\mathrm{is}\;\mathrm{calculated}\;\mathrm{outside}\;\mathrm{the}\;\mathrm{model}$$

(9) $$\delta \;∼\;\mathrm{normal}\left(\delta^{*},\;\frac{{|\delta }^{*}|\;}{5}\right);\;\mathrm{where}\;\delta^{*}\;\mathrm{is}\;\mathrm{calculated}\;\mathrm{outside}\;\mathrm{the}\;\mathrm{model}$$

**Scaling the mean for sequencing depth:** The differences in sequencing depth across biological replicates are modelled with a (log-)scaling factor ϵ that multiplies the transcript's expected abundance (mean). The scaling factor is given as known, calculated from the trimmed mean of M values (TMM) (Robinson and Oshlack 2010) and the library size [as calculated by tidybulk (Mangiola *et al.* 2021a)]. This scaling principle is also used through diverse approaches by some popular methods for differential gene-transcript abundance, such as DESEeq2 (Love *et al.* 2014), edgeR (McCarthy *et al.* 2012), and ppcseq (Mangiola *et al.* 2021b).


(10) $$Y_{s,g}∼\mathrm{NegativeBinomial}\;\left(\;\text{exp}\;\left(X_{s,g}\alpha_{c,g}+Z_{s,r}\beta_{r,g}+\epsilon_{s}\right),\;\text{exp}\;\left(\sigma_{g}\right)\right)$$

**Core model:** The data is modelled as generated from a Negative Binomial distribution with the above parameters [Equation (10)].

**Mean prior:** The expected log abundance *α* across genes is modelled as generated by a skewed normal distribution, where μ represents the mean, *ψ* represents the standard deviation and v the skewness [Equation (11)]. These parameters have hyperpriors defined by variables set outside the model, informed by the datasets analyzed in this study, and can alternatively be set by the user [Equations (12–14)].


(11) $$\alpha \;∼\;\mathrm{skewNormal}\left(\mu ,\;\exp\left(\psi \right),\;\upsilon \right)$$

(12) $$\mu \;∼\;\mathrm{normal}\left(\mu^{*},\;\frac{{|\mu }^{*}|\;}{5}\right);\;\mathrm{where}\;\mu^{*}\;\mathrm{is}\;\mathrm{calculated}\;\mathrm{outside}\;\mathrm{the}\;\mathrm{model}$$

(13) $$\psi \;∼\;\mathrm{normal}\left(\psi^{*},\;\frac{{|\psi }^{*}|\;}{5}\right);\;\mathrm{where}\;\psi^{*}\;\mathrm{is}\;\mathrm{calculated}\;\mathrm{outside}\;\mathrm{the}\;\mathrm{model}$$

(14) $$\upsilon \;∼\;\mathrm{normal}\left(\upsilon^{*},\;\frac{{|\upsilon }^{*}|\;}{5}\right);\;\mathrm{where}\;\upsilon^{*}\;\mathrm{is}\;\mathrm{calculated}\;\mathrm{outside}\;\mathrm{the}\;\mathrm{model}$$

**Inference:** This set of sampling statements and the data is provided to Stan (Carpenter *et al.* 2017) to sample from a joint posterior distribution of the model parameters [Equation (15)]. Stan uses a dynamic Hamiltonian Monte Carlo sampling algorithm, a variation on the Markov-chain Monte Carlo sampling method. By default, four Markov chains are run. The number of burn-in iterations is 300 for each chain, and the number of sampling iterations is 500 per chain, giving a base of 50 draws for the 2.5% and 97.5% quantiles.


(15) $$\begin{array}{c} P\left(\lambda_{0},\lambda_{1},\delta ,\mu ,\psi ,\upsilon ,\omega ,ο\right)\prod_{g=1}^{G}P\left(\sigma_{g}|\alpha_{g},\lambda_{0},\lambda_{1},\delta \right)\;P\left(\rho_{g}|\omega ,o\right)\\ \;\prod_{g=1}^{G}\prod_{c=1}^{C}P\left(\alpha_{g,c}|\mu ,\phi ,\upsilon \right)\prod_{g=1}^{G}\prod_{r=1}^{R}P\left(\beta_{g,c}|\omega ,o\right) \end{array}$$

### 2.3 Data missingness

A missing data point is a gene missing from a set of samples, for which the missingness cannot be attributed to being zero-count. Our model naturally allows for data missingness. At the estimation time, sample/gene pairs are fitted against the parameters by Stan, sequentially. Missing data points are simply omitted from the fitting process.

### 2.4 Generation of adjusted data

To obtain study-effect-free transcriptome datasets, cellsig generates data from the fitted posterior distribution (posterior-predictive simulation) only from the population-level (i.e. fixed) cell-type effects. We generate the same number of samples as the raw data for consistency. As a form of regularization, we generate data within the 80% credible interval of the marginal distributions of each sample-transcript pair.

### 2.5 Data analysis

To model the transcript-abundance distribution for a particular cell type with many heterogeneous samples from diversified data sources, we apply cellsig on raw data and provide cellsig-adjusted data to signature selection methods (i.e. CIBERSORTx in this study). This approach was applied to 23 of the most common immune and nonimmune cell types within the human bulk transcriptional catalogue (HBCC), covered by a large number of transcriptome datasets. We selected endothelial cells, epithelial cells, fibroblast cells, mast cells, memory B cells, naïve B cells, eosinophil, monocyte, neutrophil, CD56^bright^ NK cells, CD56^dim^ NK cells, γδ-T cells, immature myeloid dendritic cells, mature myeloid dendritic cells, M1-macrophages, M2-macrophages, naïve CD8^+^ T-cells, helper T-cells, regulatory T-cells, central CD4^+^ memory T-cells, effector CD4^+^ memory T-cells, central CD8^+^ memory T-cells, and effector CD8^+^ memory T-cells.

For this data, cellsig estimated the heterogeneity for cell-type transcriptomes, modelling population (i.e. fixed) and group (i.e. random; e.g. study) effects. We organized cell types into a differentiation hierarchy. For each node of the hierarchy, cellsig allows for missing information due to partial gene overlap across samples (e.g. missing gene-sample pairs). The generated dataset is then input to CIBERSORTx to generate the transcriptional signatures.

### 2.6 Cell-type-specific feature selection

We utilized the signature generation module of CIBERSORTx (Newman *et al.* 2019). This algorithm identifies cell-type-specific marker genes by comparing the transcriptomes of multiple cell types, producing a transcriptional signature matrix of the cell types that can be used for the deconvolution of bulk-tissue transcriptomes. We utilized this tool to generate transcriptional signature matrices for 23 different cell types (see subsection Data analysis). We used two input datasets for the signature generation; (i) the dataset containing raw transcriptomes and (ii) a simulated transcriptome dataset with transcript abundances estimated from the Bayesian modelling. For the raw count dataset, the number of genes is inconsistent across these cell types, so we first selected only those genes present across all 23 cell types in at least 1 sample. Then we imputed the missing transcript abundances for each of the samples of a given cell type. As for the simulated dataset from Bayesian modelling, we followed the workflow described in the previous section.

For both dataset types, we prepared transcriptional signature matrices with varying sizes (50, 100, 200, 500, 1000, and 2000 markers) by adjusting the G.min and G.max parameters of CIBERSORTx (signatures and the specific parameters are provided in Supplementary Table S3). For both input types and all the different signatures, the *q*-value parameter was always specified to 0.01, and the rest of the other parameters were kept default.

### 2.7 Deconvolution benchmark

We evaluated the performance of the adjusted signatures in deconvolving artificial tissue transcriptomes with known composition. Signatures that lead to a small error of the estimated cell-type proportions from their true proportions are deemed better in deconvolution. One hundred *in silico* mixtures were simulated by *in silico* mixtures with known proportions randomly generated from Dirichlet distributions. These *in silico* mixtures were prepared using the cellsig package (generate_mixture_from_proportion_matrix function). These randomly generated proportions were used as the ground truth. We benchmarked the ability of the cell-type signatures estimated with CIBERSORTx from raw or adjusted data to produce accurate deconvolution. We used three deconvolution methods: CIBERSORT (Newman *et al.* 2015), EPIC (Racle and Gfeller 2020), and linear least-square regression (LLSR) (Abbas *et al.* 2009) implemented in the tidybulk package (deconvolve_cellularity function). Finally, the mean-absolute-error values were obtained for each signature from the deconvolution of a transcriptome mixture data with the following equation.


$$\mathrm{Mean}\;\mathrm{absolute}\;\mathrm{error}=\frac{\sum \left|(\mathrm{Reference}\;\mathrm{proportion}\;\mathrm{of}\;a\;\mathrm{cell}\;\mathrm{type}-\mathrm{Estimated}\;\mathrm{proportion}\;\mathrm{of}\;\mathrm{that}\;\mathrm{cell}\;\mathrm{type})\right|}{\mathrm{Total}\;\mathrm{number}\;\mathrm{of}\;\mathrm{cell}\;\mathrm{types}}$$

In addition, we used the Philentropy package (Drost 2018) to calculate the Jensen–Shannon divergences (Lin 1991) from the estimated and ground truth proportions. To assess the deconvolution accuracy, we calculated the Pearson correlation coefficients (Benesty *et al.* 2009) between the ground truth and the estimated cell proportions. To provide another line of evidence about the performances of cellsig, we evaluated the cell-type specificity of each marker to one cell type preferentially (i.e. a gene marker highly transcribed in one cell type and lowly transcribed in all the others). First, we ranked the cell types for each marker based on descending transcriptional abundance. Next, we compared the fold difference in transcriptional abundances between the first and the second cell type, which we referred to as ‘second-fold-change’ (Fig. 1).

### 2.8 User interface

To allow visualization of the catalogue and the Bayesian estimates of transcriptional profiles, we developed an interactive web application using RShiny v1.7.2 running on R v4.2.0. The first tab in the application allows for visual inspection of the gene’s relative abundances across 23 cell types in the form of violin plots. The transcript-abundance level is represented as a log10-transformed version of TMM-scaled-counts. The estimates of the transcript abundances are represented by a red quantile bar overlayed on top of the violin plots. The lower and upper bars represent the 80% credible intervals of the Bayesian-estimated abundances. Also, the cell-type-cluster-specific transcript-abundance patterns of the query gene can be visualized through the PCA plot. This PCA clustering of the samples has been done based on the identified markers from the Bayesian simulated-1000 signature described in the previous section. The top-left PCA plot allows the cluster-specific visualization of the transcript abundances, while the top-right PCA plot guides the cell clusters. The second tab lets users download the database.

## 3 Results and discussion

### 3.1 The HBCC catalogue harmonizes 1435 bulk samples across 67 cell types

To create a comprehensive and harmonized data source, we curated the cell type annotation of the HBCC catalogue, which includes RNA sequencing samples from 58 studies, using a cell differentiation ontology spanning 67 cell types. Of these, 49 represent differentiation endpoints (e.g. CD8 Memory T cells), and 18 nodes represent cellular differentiation intermediates (e.g. T cell) (Supplementary Fig. S1).

**Figure 2.**
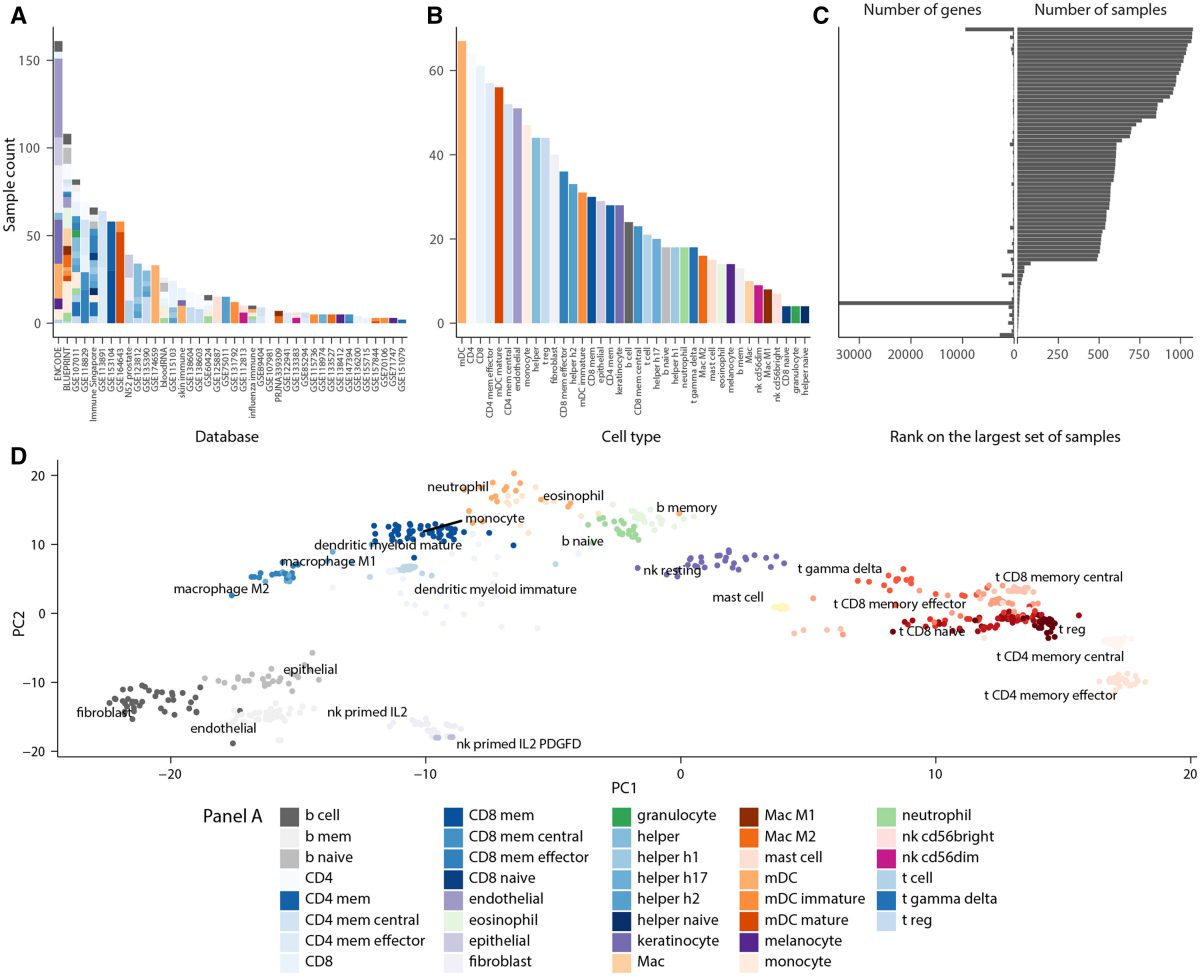
Summary of the HBCC database. Bar plots showing the (A) sample-to-datasets and (B) samples-to-cell-type distribution variations. Each coloured bar indicates a cell type. (C) Bidirectional bar plot showing the variation of library sizes across the included samples with the database. (D) PCA plot with the identified markers of 24 selected cell types representing distinct sample-clustering for each cell type.

Across all datasets, 72% of samples were isolated from the tissue by flow sorting; 28% were primary cultures (including *in vitro* differentiated cells). Of those samples, only controls (untreated or unstimulated) were included. Samples derived from cell lines were excluded. Overall, 194 highly correlated (*R*-square > 0.99) samples were excluded to avoid experimental duplicates and studies with extremely low variability. For each cell type, we harmonized the transcriptome samples from several datasets (Fig. 2A and B, Table 1).

After filtration and curation, the catalogue included 1435 samples, of which 1006 were annotated at the higher-order nodes of the cell-developmental hierarchy outlining the broad generic cell types (e.g. T and B cells). In contrast, the remaining 429 samples belong to the more branched nodes for fully differentiated cell subsets (e.g. CD8 effector memory T cells) (Supplementary Fig. S1). The library size (i.e. total gene-transcript count per sample) ranged from 706 to 63 925 across samples, with a median of 27 237.

Due to the diversity of the data sources, the set of genes with transcript-abundance information varied across samples. The nature of gene missingness cannot be easily identified for heterogeneous catalogues. Therefore, we adopted the parsimonious approach of treating genes with no transcript-abundance information as missing data, leaving the estimation to be influenced by observed information. A total of 739 genes are shared across 90% of samples, and 20 878 genes are shared across 50% of samples (Fig. 2C).

### 3.2 The web interface of the HBCC catalogue facilitates interactive gene marker exploration

**Figure 3.**
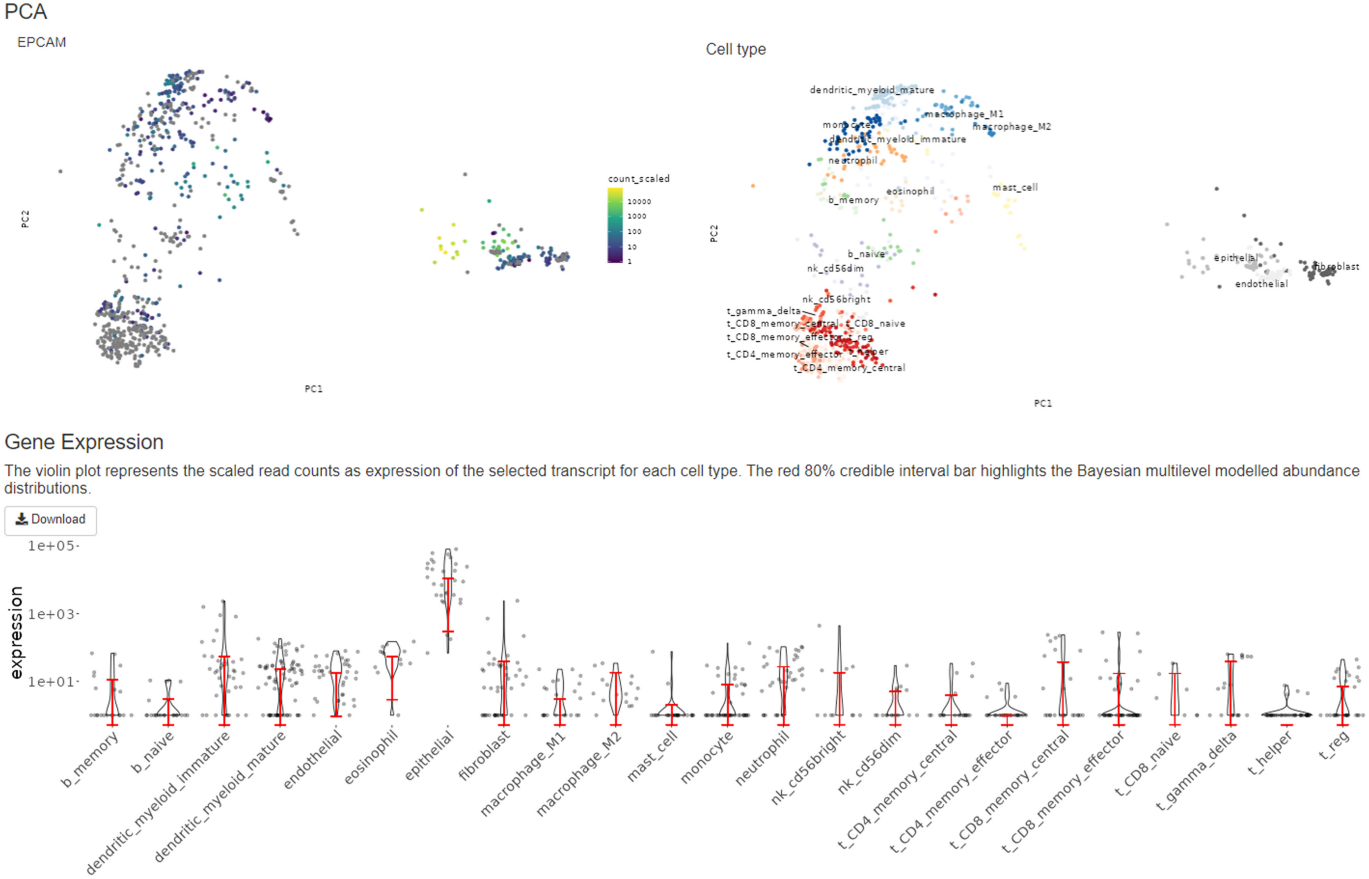
The interactive web interface of HBCC. Raw and Bayesian-modelled transcript-abundance distribution of the EPCAM gene across 23 different cell types are presented here. Top-left PCA plot highlights the expression of this gene in different cell-type clusters where luminosity indicates expression levels, dots that are not colour-coded represent the samples which do not have any expression information for the query transcript.

To enable easy access and navigation of the HBCC database, we developed a Shiny-based interactive web interface (available at github.com/stemangiola/cellsig/). This interface allows downloading the entire HBCC database in CSV format and provides an interactive platform for exploring gene markers. Through the gene-expression-comparison tab, users can visualize the abundance distribution of a particular gene across different cell types using violin and principal component analysis (PCA) plots of the clustered samples (Fig. 3). Comparisons of transcript abundances for 21 714 genes across cell types can be visualized in this tab. Users can select a gene of interest from the drop-down selection and focus on the count distribution in the immune or nonimmune cell clusters by choosing the appropriate cellular compartment.

The violin plots are generated from the scaled read counts of the selected transcript for each cell type and overlaid with the adjusted abundance distributions for the selected gene, represented by a red-coloured 80% credible interval bar of the modelled abundance. Users can also download the complete HBCC database as a compressed RDS file via the ‘Download Dataset’ tab or retrieve the harmonized transcriptome datasets for a specific cell type. The downloadable datasets include raw transcript abundances, sample identifiers, data sources, and designated cell-type information. This web interface provides an intuitive and user-friendly tool for experimental and computational biologists to explore and utilize the HBCC data for their research.

### 3.3 The sparse-information random-intercept model accurately estimates transcription variability on heterogeneous catalogues

**Figure 4.**
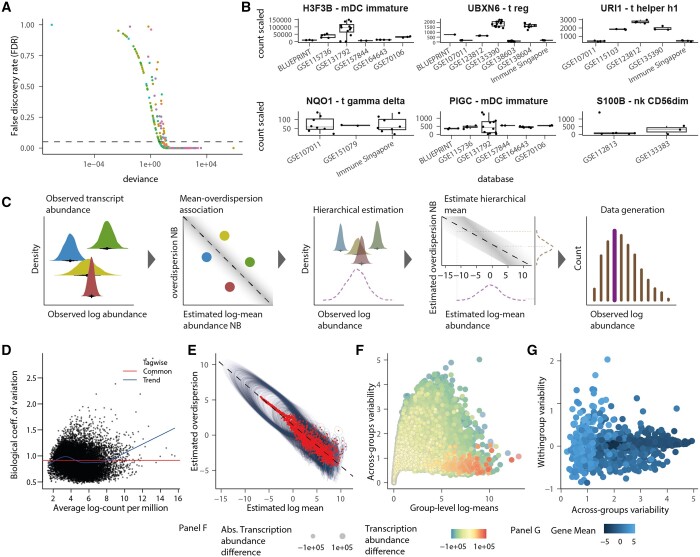
Random intercept model multisource catalogue. (A) Significance of ANOVA test (on negative binomial generalized linear model) of each gene/cell type across datasets. Each dot is a gene/cell-type pair, coloured by cell type. The dashed line represents the significance threshold of 0.05 false-discovery rate. (B-top) A selection of three genes/cell-type pairs representing significant clustering according to the dataset. (B-bottom) A selection of three pairs in which transcript abundance is consistent across datasets. (C) Cartoon of the Bayesian random intercept model estimates gene/cell-type transcript abundance representing one gene-cell-type pair and four datasets. The four bright densities are the observed data distributions. The four bright dots are the point estimates. The dark distributions are the posterior densities for the estimates group –log mean transcript abundance. The purple dashed line is the posterior density for the group-level log-mean transcript abundance; the brown dashed density is the group-level standard deviation of transcript abundance. The thick line in the histogram represents the mean generated data distribution informed by the log-mean posterior density. In contrast, the thin lines represent the part of the generated data distribution informed by the overdispersion posterior density. (D) The edgeR trend of the tag-wise dispersion, on which estimation shrinkage is based. (E) The association between log-mean and log-overdispersion is linear compared to the association between log count per million and coefficient of variation modelled by edgeR (D). Red dots are point estimates, and the ellipse represents the uncertainty described by the posterior distribution (95% credible interval). (F) Marker genes (red-shaded points) have high transcription (*x*-axis) and low variability across datasets (*y*-axis, for the comparison of endothelial versus immune, fibroblasts, and epithelial). (G) The variability of gene-transcript abundance across datasets (*x*-axis) and within datasets (*y*-axis) are not associated (for endothelial cells).

Modelling gene-transcript abundance from multiple data sources without a consistent experimental design poses unique challenges that require a tailored and flexible model to represent the hierarchy of samples within studies. Standard transcriptomic analyses focus on variability across samples; however, in a multisource catalogue, the dominant variability is across studies (Fig. 4A). For some genes, the mean transcription is consistent across studies (Fig. 4B, bottom panel), and the variability dominates at the sample level, whereas for most genes (Figs 4A and 3B, top panel), the variability dominates at the study level.

To address this issue and produce accurate estimates compatible with missing data and hierarchical data structure (e.g. samples within studies/datasets), we developed a Bayesian sparse multilevel model tailored to RNA sequencing data (Fig. 4C). For each gene, our model estimates the mean transcript abundance of each study, modelling samples as repeated observations (Fig. 4C, first subpanel). The variability for a gene-study pair is modelled separately under a hierarchical prior. The mean-variability association, typical of RNA sequencing data, is modelled in the log space at the population level (i.e. fixed effect) (Fig. 4C, second subpanel). The group-level (i.e. across datasets) transcriptional abundance is estimated as the mean across studies, with each study representing an independent observation (Fig. 4C, third subpanel). The group-level gene variability is estimated by mapping the group-level gene transcript abundance to the mean-variability association (Fig. 4C, fourth subpanel). A theoretical data distribution is drawn from the joint parameter distribution, representing the generative data process underlying the multilevel observed data source (Fig. 4C, fifth subpanel).

Compared to the mean-variability association estimated on the observed counts by several frequentist methods (Introduction section; Fig. 4D), our approach has the advantages of (i) hierarchically modelling the association in the latent space between estimated means and variabilities, (ii) using log space, and (iii) parameterizing gene variability as overdispersion. This approach allows for identifying a linear relationship (Mangiola *et al.* 2021a,b) (Fig. 4E) without gene filtering (Law *et al.* 2016). The group-level estimates and their uncertainties make it possible to identify the differential abundance and cell-type marker ranking based on quantiles without standard hypothesis testing. For example, suitable upregulated marker genes can be identified among the genes with low variability and high mean abundance for endothelial cells compared with epithelial, fibroblasts, and immune cells (Fig. 4F). Ideally, marker genes for a cell type are characterized by a low within- and across-group variability (Fig. 4G).

### 3.4 Sparse, multilevel modelling enhances cell-type signature estimation

To evaluate the benefits of using our Bayesian modelling in the cell-type signature selection, we tested CIBERSORTx signature estimation using raw data and cellsig-adjusted data. CIBERSORTx provides a robust pipeline for generating transcriptional signatures (with gene marker selection) from replicated bulk cell-type transcriptomes (Newman *et al.* 2019). Considering that the self-similarity of samples from the same dataset and the unbalanced dataset size could bias the signature estimation process, we sought to determine whether modelling and adjusting for these effects improves signature estimation. Specifically, we tested if adjusting the input data (for the CIBERSORTx signature estimation algorithm) for study-level effects and imbalance between study size (using multilevel modelling), allowing for missing information (i.e. sample-gene pairs) can improve the cell-type signatures, the marker-gene ranking, and the deconvolution accuracy from those signatures.

**Figure 5.**
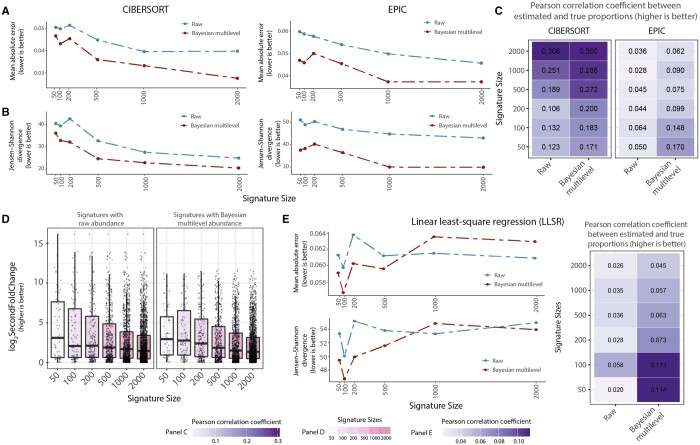
cellsig improves the CIBERSORTx estimation of cell-type transcriptomic signatures with multilevel transcriptional adjustment. (A) Benchmark of CIBERSORTx-generated transcriptional signatures with varying sizes from raw or adjusted transcript abundance. The deconvolution accuracy (for CIBERSORTx and EPIC) was measured with the median (across 100 in silico mixtures) mean-absolute-error values. (B) The deconvolution accuracy was measured with Jensen–Shannon divergence. (C) Pearson’s correlation coefficient values between the ground truth and estimated proportions from the deconvolutions. (D) Boxplots of second-fold-change scoring of the signatures generated by CIBERSORTx. The second-fold-change scores the difference in abundance of the markers between cell types where they are expressed highest and second highest. Boxplots are arranged and colour-coded for signature sizes. (E) Deconvolution benchmark using llsr from signatures generated by CIBERSORTx. (F) Pearson's correlation coefficient between the ground truth and estimated proportions from the llsr deconvolution method.

We estimated the signature from the raw and adjusted data and compared the deconvolution accuracy across popular deconvolution methods that allow for custom input signatures, including CIBERSORT (support vector regression) and EPIC (constrained linear regression). cellsig provided a significant performance improvement for the adjusted input transcriptomes across signature sizes (*n* = 50, 100, 200, 500, 1000, and 2000) and deconvolution methods (Fig. 5A–C). The second-fold-change scoring showed the highest enrichment in robust cell-type-specific markers in the top marker rank after cellsig adjustment (Fig. 5D, Supplementary Fig. S2C). This better gene-marker ranking likely contributed to the improved deconvolution accuracy.

For comparative purposes, we tested the differences in deconvolution accuracy using a vanilla, outlier-sensitive deconvolution method, such as the linear least-square regression (LLSR). The adjusted reference performs significantly better than the raw reference for small signature sizes.

The raw reference shows no significant association between deconvolution accuracy and signature size (a proxy of gene rank); on the contrary, the adjusted reference shows a negative association (Fig. 5E). This finding suggests that the most informative marker genes are concentrated in the top gene ranks and increasing the signature size beyond 500 increases the noise-to-information ratio, which affects the accuracy of llsr. Although the effect is noticeable, the difference in accuracy is limited due to our highly heterogeneous training and test sets (including 829 samples, Supplementary Fig. S3).

## 4 Conclusion

In recent years, the scientific community has produced a vast amount of single-cell transcriptomes, including the Human Cell Atlas (Osumi-Sutherland *et al.* 2021), and bulk, experimentally purified transcriptomes such as ENCODE and ad hoc studies (ENCODE Project Consortium 2012, Abugessaisa *et al.* 2016, Fernández *et al.* 2016). This data richness creates unprecedented opportunities to estimate cell-type transcriptomics profiles; however, it poses challenges due to data heterogeneity. The transcriptional heterogeneity is especially present across studies, for which datasets are often unbalanced in size, biasing the signature estimation process.

Addressing these unique challenges, our Bayesian model provides a novel approach to estimating cell-type transcriptional signatures, adjusting the raw data before using signature estimation and marker-gene selection methods such as CIBERSORTx. Our models sample-level and study-level variability from sparse data (i.e. nonoverlapping gene sets across samples) and can generate data from the study-level-free underlying distribution. Our benchmark showed the efficacy of cellsig to improve CIBERSORTx gene ranking, leading to downstream improved deconvolution accuracy.

Our benchmark was performed on a novel, large-scale, curated human bulk transcriptional catalogue that harmonizes 1435 samples across 58 datasets and 67 cell types. We share this catalogue with the community through a web Shiny interface to explore gene markers and visualize gene-expression comparisons. Such an interface bridges the gap between this vast dataset and biologists, facilitating easier data extraction and visualization.

In an era where vast data enables comprehensive profiling of human-tissue cell types, cellsig emerges as a useful tool for scaling up signature estimation, impacting tissue deconvolution, single-cell annotation, and marker-gene validation.

## Supplementary Material

btad685_Supplementary_Data

## Data Availability

All data included in this study were obtained from publicly available sources (Table 1). cellsig is implemented as an R package (Github: stemangiola/cellsig). Code used to generate figures and perform analyses can be found at https://github.com/stemangiola/cellsig.
