# Supplementary material for: cellsig plug-in enhances CIBERSORTx signature selection for multidataset transcriptomes with sparse multilevel modelling
Source: Bioinformatics. 2023 Nov 11;39(12):btad685. doi: 10.1093/bioinformatics/btad685 (PMC10692870; doi:10.1093/bioinformatics/btad685)
Supplement: btad685_Supplementary_Data [file btad685_supplementary_data.zip › Table S4.docx]

**Table S4.** **Datasets included in the HBCC database.**

| **Name** | **Samples** | **Cell types** | **Reference** |
| --- | --- | --- | --- |
| ENCODE | 382 | 25 | [(Luo *et al.*, 2020)](https://paperpile.com/c/C1at2t/Hd9kS) |
| BLUEPRINT | 84 | 29 | [(Fernández *et al.*, 2016)](https://paperpile.com/c/C1at2t/u7K6M) |
| GSE107011 | 106 | 19 | [(Monaco *et al.*, 2019)](https://paperpile.com/c/C1at2t/8meMV) |
| GSE118829 | 69 | 6 | [(Takeshita *et al.*, 2019)](https://paperpile.com/c/C1at2t/fStNl) |
| GSE113891 | 64 | 2 | [(da Silva Antunes *et al.*, 2018)](https://paperpile.com/c/C1at2t/orVda) |
| GSE164643 | 36 | 2 | [(Kroes *et al.*, 2021)](https://paperpile.com/c/C1at2t/XnjnG) |
| GSE153104 | 47 | 2 | [(Dhanwani *et al.*, 2020)](https://paperpile.com/c/C1at2t/rggBA) |
| EGAD00001004948 | 52 | 4 | [(Mangiola *et al.*, 2021)](https://paperpile.com/c/C1at2t/wXYw0) |
| GSE77808 | 28 | 2 | [(Mao *et al.*, 2016)](https://paperpile.com/c/C1at2t/WoDQ4) |
| GSE123812 | 38 | 6 | [(Yost *et al.*, 2019)](https://paperpile.com/c/C1at2t/7st5h) |
| GSE115898 | 33 | 4 | [(Ahn *et al.*, 2017)](https://paperpile.com/c/C1at2t/fiZmh) |
| GSE135390 | 32 | 6 | [(Höllbacher *et al.*, 2020)](https://paperpile.com/c/C1at2t/lt5iY) |
| GSE174659 | 29 | 1 | [(Lepzien *et al.*, 2021)](https://paperpile.com/c/C1at2t/3zvj3) |
| GSE115103 | 25 | 4 | [(Loyal *et al.*, 2020)](https://paperpile.com/c/C1at2t/KpWXa) |
| bloodRNA | 26 | 7 | NA |
| GSE125887 | 13 | 1 | [(Cildir *et al.*, 2019)](https://paperpile.com/c/C1at2t/rs2yV) |
| GSE60424 | 17 | 6 | [(Linsley *et al.*, 2014)](https://paperpile.com/c/C1at2t/Y4Nq0) |
| GSE138604 | 13 | 2 | [(Mensink *et al.*, 2022)](https://paperpile.com/c/C1at2t/PKJK2) |
| GSE138603 | 16 | 2 | [(Mensink *et al.*, 2022)](https://paperpile.com/c/C1at2t/PKJK2) |
| GSE75011 | 15 | 1 | [(Seumois *et al.*, 2016)](https://paperpile.com/c/C1at2t/bCW8m) |
| GSE122324 | 12 | 2 | [(Wang *et al.*, 2020)](https://paperpile.com/c/C1at2t/Bzm0g) |
| GSE131792 | 11 | 1 | [(Srivastava *et al.*, 2020)](https://paperpile.com/c/C1at2t/F0F5R) |
| GSE145450 | 11 | 2 | [(Agarwal *et al.*, 2020)](https://paperpile.com/c/C1at2t/5TEXz) |
| GSE89404 | 11 | 3 | [(Barski *et al.*, 2017)](https://paperpile.com/c/C1at2t/VvcCU) |
| GSE64655 | 11 | 6 | [(Hoek *et al.*, 2015)](https://paperpile.com/c/C1at2t/3Yo1L) |
| PRJNA339309 | 12 | 3 | [(Lurier *et al.*, 2017)](https://paperpile.com/c/C1at2t/Nt04b) |
| GSE130379 | 10 | 1 | [(Marquardt *et al.*, 2019)](https://paperpile.com/c/C1at2t/9xqvz) |
| GSE138412 | 5 | 1 | [(Hanniford *et al.*, 2020)](https://paperpile.com/c/C1at2t/UrxbL) |
| GSE85294 | 8 | 3 | [(Spurlock *et al.*, 2017)](https://paperpile.com/c/C1at2t/NOKjl) |
| GSE107047 | 8 | 2 | [(Barrow *et al.*, 2018)](https://paperpile.com/c/C1at2t/JQ3jG) |
| GSE101396 | 8 | 2 | [(Zaal *et al.*, 2017)](https://paperpile.com/c/C1at2t/cNUph) |
| GSE107981 | 8 | 1 | [(Abadier *et al.*, 2017)](https://paperpile.com/c/C1at2t/E4dtJ) |
| GSE147394 | 8 | 2 | [(Galletti *et al.*, 2020)](https://paperpile.com/c/C1at2t/lDTam) |
| GSE115736 | 7 | 2 | [(Choi *et al.*, 2019)](https://paperpile.com/c/C1at2t/RGRZD) |
| GSE128027 | 7 | 1 | [(Nelson *et al.*, 2019)](https://paperpile.com/c/C1at2t/ZjQm1) |
| GSE122941 | 2 | 1 | [(Emming *et al.*, 2020)](https://paperpile.com/c/C1at2t/4KqP8) |
| GSE133383 | 6 | 2 | [(Dogra *et al.*, 2020)](https://paperpile.com/c/C1at2t/F3ch0) |
| GSE118974 | 5 | 1 | [(Tripathi *et al.*, 2019)](https://paperpile.com/c/C1at2t/PzaDD) |
| GSE125916 | 5 | 1 | [(Mack *et al.*, 2020)](https://paperpile.com/c/C1at2t/pxzaQ) |
| GSE133527 | 5 | 1 | [(Quero *et al.*, 2019)](https://paperpile.com/c/C1at2t/GaeOi) |
| GSE107316 | 4 | 1 | NA |
| GSE122325 | 4 | 2 | [(Wang *et al.*, 2020)](https://paperpile.com/c/C1at2t/Bzm0g) |
| GSE136200 | 4 | 1 | [(Khan *et al.*, 2020)](https://paperpile.com/c/C1at2t/yZlQj) |
| GSE141410 | 4 | 1 | NA |
| GSE152571 | 4 | 1 | [(Wu *et al.*, 2020)](https://paperpile.com/c/C1at2t/SJvWm) |
| GSE157844 | 4 | 2 | [(Scheenstra *et al.*, 2020)](https://paperpile.com/c/C1at2t/bpnaY) |
| GSE89087 | 4 | 2 | [(Götz *et al.*, 2017)](https://paperpile.com/c/C1at2t/lar6i) |
| GSE130286 | 3 | 1 | [(Sabry *et al.*, 2019)](https://paperpile.com/c/C1at2t/kjTUK) |
| GSE133478 | 3 | 1 | [(Wagstaffe *et al.*, 2019)](https://paperpile.com/c/C1at2t/KuioF) |
| GSE137229 | 3 | 1 | [(Arora *et al.*, 2020)](https://paperpile.com/c/C1at2t/5Pacm) |
| GSE146028 | 3 | 1 | [(Gurvich *et al.*, 2020)](https://paperpile.com/c/C1at2t/Wb63Q) |
| GSE146867 | 3 | 1 | [(Alqassim *et al.*, 2021)](https://paperpile.com/c/C1at2t/4mhnx) |
| GSE155715 | 3 | 1 | [(Foskolou *et al.*, 2020)](https://paperpile.com/c/C1at2t/Fl3eW) |
| GSE70106 | 3 | 1 | [(Kumar *et al.*, 2015)](https://paperpile.com/c/C1at2t/GEepS) |
| GSE71747 | 2 | 1 | [(Jin *et al.*, 2015)](https://paperpile.com/c/C1at2t/9p9va) |
| GSE144990 | 2 | 1 | [(Hu *et al.*, 2021)](https://paperpile.com/c/C1at2t/zPbcJ) |
| GSE151079 | 2 | 1 | [(Roels *et al.*, 2020)](https://paperpile.com/c/C1at2t/YoYlR) |
| GSE165804 | 2 | 1 | [(Dwyer *et al.*, 2021)](https://paperpile.com/c/C1at2t/K7ZTE) |

# References

[Abadier,M. *et al.* (2017) Effector and Regulatory T Cells Roll at High Shear Stress by Inducible Tether and Sling Formation. *Cell Rep.*, **21**, 3885–3899.](http://paperpile.com/b/C1at2t/E4dtJ)

[Agarwal,S. *et al.* (2020) The long non-coding RNA LUCAT1 is a negative feedback regulator of interferon responses in humans. *Nat. Commun.*, **11**, 6348.](http://paperpile.com/b/C1at2t/5TEXz)

[Ahn,R.S. *et al.* (2017) Transcriptional landscape of epithelial and immune cell populations revealed through FACS-seq of healthy human skin. *Sci. Rep.*, **7**, 1343.](http://paperpile.com/b/C1at2t/fiZmh)

[Alqassim,E.Y. *et al.* (2021) RNA editing enzyme APOBEC3A promotes pro-inflammatory M1 macrophage polarization. *Commun Biol*, **4**, 102.](http://paperpile.com/b/C1at2t/4mhnx)

[Arora,P. *et al.* (2020) Body fluid from the parasitic worm Ascaris suum inhibits broad-acting pro-inflammatory programs in dendritic cells. *Immunology*, **159**, 322–334.](http://paperpile.com/b/C1at2t/5Pacm)

[Barrow,A.D. *et al.* (2018) Natural Killer Cells Control Tumor Growth by Sensing a Growth Factor. *Cell*, **172**, 534–548.e19.](http://paperpile.com/b/C1at2t/JQ3jG)

[Barski,A. *et al.* (2017) Rapid Recall Ability of Memory T cells is Encoded in their Epigenome. *Sci. Rep.*, **7**, 39785.](http://paperpile.com/b/C1at2t/VvcCU)

[Choi,J. *et al.* (2019) Haemopedia RNA-seq: a database of gene expression during haematopoiesis in mice and humans. *Nucleic Acids Res.*, **47**, D780–d785.](http://paperpile.com/b/C1at2t/RGRZD)

[Cildir,G. *et al.* (2019) Genome-wide Analyses of Chromatin State in Human Mast Cells Reveal Molecular Drivers and Mediators of Allergic and Inflammatory Diseases. *Immunity*, **51**, 949–965.e6.](http://paperpile.com/b/C1at2t/rs2yV)

[Dhanwani,R. *et al.* (2020) T Cell Responses to Neural Autoantigens Are Similar in Alzheimer’s Disease Patients and Age-Matched Healthy Controls. *Front. Neurosci.*, **14**, 874.](http://paperpile.com/b/C1at2t/rggBA)

[Dogra,P. *et al.* (2020) Tissue Determinants of Human NK Cell Development, Function, and Residence. *Cell*, **180**, 749–763.e13.](http://paperpile.com/b/C1at2t/F3ch0)

[Dwyer,D.F. *et al.* (2021) Human airway mast cells proliferate and acquire distinct inflammation-driven phenotypes during type 2 inflammation. *Sci Immunol*, **6**.](http://paperpile.com/b/C1at2t/K7ZTE)

[Emming,S. *et al.* (2020) A molecular network regulating the proinflammatory phenotype of human memory T lymphocytes. *Nat. Immunol.*, **21**, 388–399.](http://paperpile.com/b/C1at2t/4KqP8)

[Fernández,J.M. *et al.* (2016) The BLUEPRINT Data Analysis Portal. *Cell Syst*, **3**, 491–495.e5.](http://paperpile.com/b/C1at2t/u7K6M)

[Foskolou,I.P. *et al.* (2020) The S enantiomer of 2-hydroxyglutarate increases central memory CD8 populations and improves CAR-T therapy outcome. *Blood Adv*, **4**, 4483–4493.](http://paperpile.com/b/C1at2t/Fl3eW)

[Galletti,G. *et al.* (2020) Two subsets of stem-like CD8(+) memory T cell progenitors with distinct fate commitments in humans. *Nat. Immunol.*, **21**, 1552–1562.](http://paperpile.com/b/C1at2t/lDTam)

[Götz,A. *et al.* (2017) Atypical activation of dendritic cells by Plasmodium falciparum. *Proc. Natl. Acad. Sci. U. S. A.*, **114**, E10568–e10577.](http://paperpile.com/b/C1at2t/lar6i)

[Gurvich,O.L. *et al.* (2020) Transcriptomics uncovers substantial variability associated with alterations in manufacturing processes of macrophage cell therapy products. *Sci. Rep.*, **10**, 14049.](http://paperpile.com/b/C1at2t/Wb63Q)

[Hanniford,D. *et al.* (2020) Epigenetic Silencing of CDR1as Drives IGF2BP3-Mediated Melanoma Invasion and Metastasis. *Cancer Cell*, **37**, 55–70.e15.](http://paperpile.com/b/C1at2t/UrxbL)

[Hoek,K.L. *et al.* (2015) A cell-based systems biology assessment of human blood to monitor immune responses after influenza vaccination. *PLoS One*, **10**, e0118528.](http://paperpile.com/b/C1at2t/3Yo1L)

[Höllbacher,B. *et al.* (2020) Transcriptomic Profiling of Human Effector and Regulatory T Cell Subsets Identifies Predictive Population Signatures. *Immunohorizons*, **4**, 585–596.](http://paperpile.com/b/C1at2t/lt5iY)

[Hu,G. *et al.* (2021) High-throughput phenotypic screen and transcriptional analysis identify new compounds and targets for macrophage reprogramming. *Nat. Commun.*, **12**, 773.](http://paperpile.com/b/C1at2t/zPbcJ)

[Jin,S.G. *et al.* (2015) The DNA methylation landscape of human melanoma. *Genomics*, **106**, 322–330.](http://paperpile.com/b/C1at2t/9p9va)

[Khan,H.N. *et al.* (2020) The circular RNA landscape in specific peripheral blood mononuclear cells of critically ill patients with sepsis. *Crit. Care*, **24**, 423.](http://paperpile.com/b/C1at2t/yZlQj)

[Kroes,M.M. *et al.* (2021) Naturally circulating pertactin-deficient Bordetella pertussis strains induce distinct gene expression and inflammatory signatures in human dendritic cells. *Emerg. Microbes Infect.*, **10**, 1358–1368.](http://paperpile.com/b/C1at2t/XnjnG)

[Kumar,N.A. *et al.* (2015) The role of antigen presenting cells in the induction of HIV-1 latency in resting CD4(+) T-cells. *Retrovirology*, **12**, 76.](http://paperpile.com/b/C1at2t/GEepS)

[Lepzien,R. *et al.* (2021) Monocytes in sarcoidosis are potent tumour necrosis factor producers and predict disease outcome. *Eur. Respir. J.*, **58**.](http://paperpile.com/b/C1at2t/3zvj3)

[Linsley,P.S. *et al.* (2014) Copy number loss of the interferon gene cluster in melanomas is linked to reduced T cell infiltrate and poor patient prognosis. *PLoS One*, **9**, e109760.](http://paperpile.com/b/C1at2t/Y4Nq0)

[Loyal,L. *et al.* (2020) SLAMF7 and IL-6R define distinct cytotoxic versus helper memory CD8(+) T cells. *Nat. Commun.*, **11**, 6357.](http://paperpile.com/b/C1at2t/KpWXa)

[Luo,Y. *et al.* (2020) New developments on the Encyclopedia of DNA Elements (ENCODE) data portal. *Nucleic Acids Res.*, **48**, D882–d889.](http://paperpile.com/b/C1at2t/Hd9kS)

[Lurier,E.B. *et al.* (2017) Transcriptome analysis of IL-10-stimulated (M2c) macrophages by next-generation sequencing. *Immunobiology*, **222**, 847–856.](http://paperpile.com/b/C1at2t/Nt04b)

[Mack,M.R. *et al.* (2020) Blood natural killer cell deficiency reveals an immunotherapy strategy for atopic dermatitis. *Sci. Transl. Med.*, **12**.](http://paperpile.com/b/C1at2t/pxzaQ)

[Mangiola,S. *et al.* (2021) Transcriptome sequencing and multi-plex imaging of prostate cancer microenvironment reveals a dominant role for monocytic cells in progression. *BMC Cancer*, **21**, 846.](http://paperpile.com/b/C1at2t/wXYw0)

[Mao,Y. *et al.* (2016) IL-15 activates mTOR and primes stress-activated gene expression leading to prolonged antitumor capacity of NK cells. *Blood*, **128**, 1475–1489.](http://paperpile.com/b/C1at2t/WoDQ4)

[Marquardt,N. *et al.* (2019) Unique transcriptional and protein-expression signature in human lung tissue-resident NK cells. *Nat. Commun.*, **10**, 3841.](http://paperpile.com/b/C1at2t/9xqvz)

[Mensink,M. *et al.* (2022) TNFR2 Costimulation Differentially Impacts Regulatory and Conventional CD4(+) T-Cell Metabolism. *Front. Immunol.*, **13**, 881166.](http://paperpile.com/b/C1at2t/PKJK2)

[Monaco,G. *et al.* (2019) RNA-Seq Signatures Normalized by mRNA Abundance Allow Absolute Deconvolution of Human Immune Cell Types. *Cell Rep.*, **26**, 1627–1640.e7.](http://paperpile.com/b/C1at2t/8meMV)

[Nelson,R.K. *et al.* (2019) Human Eosinophils Express a Distinct Gene Expression Program in Response to IL-3 Compared with Common β-Chain Cytokines IL-5 and GM-CSF. *J. Immunol.*, **203**, 329–337.](http://paperpile.com/b/C1at2t/ZjQm1)

[Quero,L. *et al.* (2019) miR-221-3p Drives the Shift of M2-Macrophages to a Pro-Inflammatory Function by Suppressing JAK3/STAT3 Activation. *Front. Immunol.*, **10**, 3087.](http://paperpile.com/b/C1at2t/GaeOi)

[Roels,J. *et al.* (2020) Distinct and temporary-restricted epigenetic mechanisms regulate human αβ and γδ T cell development. *Nat. Immunol.*, **21**, 1280–1292.](http://paperpile.com/b/C1at2t/YoYlR)

[Sabry,M. *et al.* (2019) Tumor- and cytokine-primed human natural killer cells exhibit distinct phenotypic and transcriptional signatures. *PLoS One*, **14**, e0218674.](http://paperpile.com/b/C1at2t/kjTUK)

[Scheenstra,M.R. *et al.* (2020) Comparison of the PU.1 transcriptional regulome and interactome in human and mouse inflammatory dendritic cells. *J. Leukoc. Biol.*](http://paperpile.com/b/C1at2t/bpnaY)

[Seumois,G. *et al.* (2016) Transcriptional Profiling of Th2 Cells Identifies Pathogenic Features Associated with Asthma. *J. Immunol.*, **197**, 655–664.](http://paperpile.com/b/C1at2t/bCW8m)

[da Silva Antunes,R. *et al.* (2018) Th1/Th17 polarization persists following whole-cell pertussis vaccination despite repeated acellular boosters. *J. Clin. Invest.*, **128**, 3853–3865.](http://paperpile.com/b/C1at2t/orVda)

[Spurlock,C.F.,3rd *et al.* (2017) Profiles of Long Noncoding RNAs in Human Naive and Memory T Cells. *J. Immunol.*, **199**, 547–558.](http://paperpile.com/b/C1at2t/NOKjl)

[Srivastava,P. *et al.* (2020) Inhibition of LSD1 in MDS progenitors restores differentiation of CD141(Hi) conventional dendritic cells. *Leukemia*, **34**, 2460–2472.](http://paperpile.com/b/C1at2t/F0F5R)

[Takeshita,M. *et al.* (2019) Multi-dimensional analysis identified rheumatoid arthritis-driving pathway in human T cell. *Ann. Rheum. Dis.*, **78**, 1346–1356.](http://paperpile.com/b/C1at2t/fStNl)

[Tripathi,S.K. *et al.* (2019) Quantitative Proteomics Reveals the Dynamic Protein Landscape during Initiation of Human Th17 Cell Polarization. *iScience*, **11**, 334–355.](http://paperpile.com/b/C1at2t/PzaDD)

[Wagstaffe,H.R. *et al.* (2019) Influenza Vaccination Primes Human Myeloid Cell Cytokine Secretion and NK Cell Function. *J. Immunol.*, **203**, 1609–1618.](http://paperpile.com/b/C1at2t/KuioF)

[Wang,Y. *et al.* (2020) HIV-1-induced cytokines deplete homeostatic innate lymphoid cells and expand TCF7-dependent memory NK cells. *Nat. Immunol.*, **21**, 274–286.](http://paperpile.com/b/C1at2t/Bzm0g)

[Wu,C.Y. *et al.* (2020) Ascorbic Acid Promotes KIR Demethylation during Early NK Cell Differentiation. *J. Immunol.*, **205**, 1513–1523.](http://paperpile.com/b/C1at2t/SJvWm)

[Yost,K.E. *et al.* (2019) Clonal replacement of tumor-specific T cells following PD-1 blockade. *Nat. Med.*, **25**, 1251–1259.](http://paperpile.com/b/C1at2t/7st5h)

[Zaal,A. *et al.* (2017) TLR4 and C5aR crosstalk in dendritic cells induces a core regulatory network of RSK2, PI3Kβ, SGK1, and FOXO transcription factors. *J. Leukoc. Biol.*, **102**, 1035–1054.](http://paperpile.com/b/C1at2t/cNUph)
