# Supplementary material for: cellsig plug-in enhances CIBERSORTx signature selection for multidataset transcriptomes with sparse multilevel modelling
Source: Bioinformatics. 2023 Nov 11;39(12):btad685. doi: 10.1093/bioinformatics/btad685 (PMC10692870; doi:10.1093/bioinformatics/btad685)
Supplement: btad685_Supplementary_Data [file btad685_supplementary_data.zip › Table S2.docx]

**Supplementary Table 2: Summary information of the HBCC database.**

| **Cell type** | **No of datasets** | **No of samples** |
| --- | --- | --- |
| astrocyte | 4 | 1 |
| b_cell | 17 | 6 |
| b_memory | 21 | 4 |
| b_naive | 15 | 4 |
| basophil | 4 | 1 |
| chondrocyte | 3 | 1 |
| dendritic_myeloid | 65 | 24 |
| dendritic_myeloid_immature | 36 | 8 |
| dendritic_myeloid_mature | 50 | 7 |
| dendritic_plasmacytoid | 4 | 1 |
| endothelial | 63 | 17 |
| eosinophil | 11 | 3 |
| epithelial | 47 | 11 |
| erythroblast | 8 | 1 |
| fibroblast | 73 | 20 |
| granulocyte | 4 | 1 |
| keratinocyte | 36 | 12 |
| lymphoid | 5 | 1 |
| macrophage | 6 | 1 |
| macrophage_M0 | 4 | 1 |
| macrophage_M1 | 18 | 6 |
| macrophage_M2 | 11 | 3 |
| mait | 4 | 1 |
| mast_cell | 19 | 3 |
| megakaryocyte | 5 | 1 |
| melanocyte | 15 | 4 |
| monocyte | 46 | 8 |
| mononuclear | 2 | 1 |
| muscle | 10 | 3 |
| muscle_skeletal | 11 | 3 |
| muscle_smooth | 44 | 11 |
| myeloid | 21 | 3 |
| myocyte | 8 | 2 |
| natural_killer | 2 | 1 |
| neuron | 13 | 4 |
| neutrophil | 22 | 6 |
| nk_cd56bright | 11 | 3 |
| nk_cd56dim | 16 | 3 |
| nk_primed | 22 | 3 |
| nk_primed_IL2 | 18 | 3 |
| nk_primed_IL2_PDGFD | 4 | 1 |
| nk_resting | 27 | 7 |
| osteoblast | 4 | 1 |
| osteoclast | 2 | 1 |
| pericyte | 4 | 1 |
| plasma_cell | 5 | 1 |
| stem_cell | 52 | 15 |
| t_CD4 | 54 | 10 |
| t_CD4_effector | 9 | 1 |
| t_CD4_memory | 25 | 1 |
| t_CD4_memory_central | 48 | 4 |
| t_CD4_memory_effector | 51 | 5 |
| t_CD4_naive | 6 | 2 |
| t_CD8 | 58 | 11 |
| t_CD8_memory | 22 | 1 |
| t_CD8_memory_central | 23 | 4 |
| t_CD8_memory_effector | 36 | 4 |
| t_CD8_naive | 7 | 4 |
| t_cell | 21 | 4 |
| t_follicular_helper | 4 | 1 |
| t_gamma_delta | 18 | 3 |
| t_helper | 47 | 7 |
| t_helper_h1 | 18 | 5 |
| t_helper_h17 | 20 | 5 |
| t_helper_h2 | 33 | 6 |
| t_helper_naive | 4 | 1 |
| t_reg | 39 | 6 |
