# Supplementary material for: cellsig plug-in enhances CIBERSORTx signature selection for multidataset transcriptomes with sparse multilevel modelling
Source: Bioinformatics. 2023 Nov 11;39(12):btad685. doi: 10.1093/bioinformatics/btad685 (PMC10692870; doi:10.1093/bioinformatics/btad685)
Supplement: btad685_Supplementary_Data [file btad685_supplementary_data.zip › Table S1.docx]

**Supplementary Table 1: Cell-type annotation convention used in constructing HBCC.**

| **Cell-type ontology used in HBCA** | **Cell-type assigned in source dataset** |
| --- | --- |
| *astrocyte* | astrocyte |
| *b_cell* | B-cells |
|  | CD19+ B cells |
|  | CD19+ B cells |
|  | CD20+ B cells |
|  | class switched memory B cell |
|  | germinal center B cell |
|  | Plasmablasts |
| *b_memory* | Exhausted B cells |
|  | memory B cell |
|  | Non-switched memory B cells |
|  | Switched memory B cells |
|  | unswitched memory B cell |
| *b_naive* | CD38-negative naive B cell |
|  | naive B cell |
|  | Naive B cells |
| *chondrocyte* | articular chondrocyte of knee joint |
| *dendritic_myeloid* | CD11c+ mDC |
|  | CD141+ dendritic cell |
|  | CD1C+ dendritic cell |
|  | conventional dendritic cell |
|  | dendritic cell |
|  | Myeloid dendritic cells |
| *dendritic_myeloid_immature* | CD141Hi |
|  | CD1c |
|  | CD1c+ dendritic cell |
|  | CD1c+ mDC |
|  | immature conventional dendritic cell |
|  | Monocyte-derived dendritic cells |
|  | monocytes derived dendritic cells |
|  | Myeloid DC CD123- |
|  | Myeloid DC CD123+ |
|  | Stimulated monocyte-derived dendritic cells |
|  | Unstimulated monocyte-derived dendritic cells |
| *dendritic_myeloid_mature* | CD1c+ dendritic cell + LPS |
|  | mature conventional dendritic cell |
|  | Monocyte-derived dendritic cells |
|  | monocyte-derived dendritic cells stimulated with live *B. pertussis* |
|  | monocytes derived dendritic cells |
|  | Stimulated monocyte-derived dendritic cells |
| *dendritic_plasmacytoid* | Plasmacytoid dendritic cells |
| *endothelial* | endothelial cell of umbilical vein |
|  | adult endothelial progenitor cell |
|  | aortic endothelial primary whole cells |
|  | bladder microvascular endothelial cell |
|  | dermis blood vessel endothelial cell |
|  | dermis lymphatic vessel endothelial cell |
|  | dermis microvascular lymphatic vessel endothelial cell |
|  | endometrial microvascular endothelial cells |
|  | endothelial cell of coronary artery |
|  | endothelial cell of umbilical vein |
|  | endothelial cell of umbilical vein (proliferating) |
|  | glomerular endothelial cell |
|  | lung microvascular endothelial cell |
|  | mammary microvascular endothelial cell |
|  | pulmonary artery endothelial cell |
|  | vein endothelial cell |
| *eosinophil* | eosinophil |
|  | mature eosinophil |
| *epithelial* | airway epithelial cell |
|  | bronchial epithelial cell |
|  | EpCAM+ epithelial cells |
|  | epithelial cell of proximal tubule |
|  | kidney epithelial cell |
|  | mammary epithelial cell |
|  | nasal cavity respiratory epithelium epithelial cell of viscerocranial mucosa |
|  | placental epithelial cell |
|  | renal cortical epithelial cell |
|  | tracheal epithelial cell |
| *erythroblast* | erythroblast |
| *fibroblast* | bronchus fibroblast |
|  | cardiac atrium fibroblast |
|  | cardiac ventricle fibroblast |
|  | CD90+ CD31- Fibroblasts |
|  | fibroblast of arm |
|  | fibroblast of dermis |
|  | fibroblast of lung |
|  | fibroblast of skin of abdomen |
|  | fibroblast of skin of back |
|  | fibroblast of skin of scalp |
|  | fibroblast of the aortic adventitia |
|  | fibroblast of villous mesenchyme |
|  | pericardium fibroblast |
| *granulocyte* | Low-density basophils |
| *basophil* | basophils |
| *keratinocyte* | keratinocyte |
|  | Epidermal keratinocytes |
|  | foreskin keratinocyte |
|  | hair follicular keratinocyte |
|  | Keratinocyte |
|  | skin keratinocyte |
| *lymphoid* | common lymphoid progenitor |
| *macrophage* | macrophage |
| *macrophage_M0* | unactivated macrophage |
| *macrophage_M1* | inflammatory macrophage |
|  | M1 Mac |
|  | Monocyte-derived macrophages |
|  | pM1 |
| *macrophage_M2* | alternatively activated macrophage |
|  | M2 macrophages |
| *mast_cell* | CD1c+ dendritic cells |
|  | Peripheral blood-derived mast cells |
|  | Umbilical cord blood-derived mast cells |
| *megakaryocyte* | CD34-negative, CD41-positive, CD42-positive megakaryocyte cell |
| *melanocyte* | Cultured epidermal melanocytes |
|  | melanocyte of skin |
| *monocyte* | CD14+ monocytes |
|  | CD14-positive monocyte |
|  | CD14-positive, CD16-negative classical monocyte |
|  | Classical monocytes |
|  | Intermediate monocytes |
|  | monocyte |
|  | Monocytes |
|  | Non classical monocytes |
| *mononuclear* | mononuclear peripheral blood |
| *muscle* | cardiac muscle cell |
| *muscle_skeletal* | skeletal muscle myoblast |
|  | skeletal muscle myosatellite cells |
| *muscle_smooth* | aortic smooth muscle cell |
|  | aortic smooth muscle cell |
|  | bronchial smooth muscle cell |
|  | smooth muscle cell |
|  | smooth muscle cell of bladder |
|  | smooth muscle cell of the coronary artery |
|  | smooth muscle cell of the pulmonary artery |
|  | smooth muscle cell of the umbilical artery |
|  | smooth muscle cell of trachea |
|  | uterine smooth muscle cell |
| *myeloid* | CD45+CD16+ myeloid cells |
|  | common myeloid progenitor |
|  | common myeloid progenitor, CD34-positive |
|  | granulocyte monocyte progenitor cell |
| *myocyte* | LHCN-M2 |
|  | regular cardiac myocyte |
| *natural_killer* | CD56+ NK cells |
| *neuron* | bipolar spindle neuron |
|  | neural cell |
| *neutrophil* | band form neutrophil |
|  | CD15+ Neutrophils |
|  | CD3+ T cells |
|  | Low-density neutrophils |
|  | mature neutrophil |
|  | Neutrophils |
|  | segmented neutrophil of bone marrow |
| *nk_cd56bright* | CD3-CD56bright |
|  | CD56brightCD16- |
|  | CD94+CD56hi |
| *nk_cd56dim* | CD45+ CD13- CD3- CD56dim |
|  | CD56dimCD16+ |
|  | CD94+CD56dim |
|  | CD94-CD56dim |
| *nk_primed* | IL-15 treated NK cells |
| *nk_primed_IL2* | IL-2 treated NK cells |
| *nk_primed_IL2* | IL2-treated NK cells |
| *nk_primed_IL2_PDGFD* | IL2+PDGFDD treated NK |
| *nk_resting* | CD56+ cells |
|  | CD56+CD16+CD57-NKG2A+ |
|  | cytotoxic CD56-dim natural killer cell |
|  | Natural killer cells |
|  | NK |
|  | NKG2A+CD16-CD69+CD49a+CD103- |
|  | NKG2A+CD16-CD69+CD49a+CD103- |
|  | NKG2A+CD16-CD69+CD49a+CD103+ |
|  | NKG2A+CD16-CD69+CD49a-CD103- |
|  | NKG2A+CD16-CD69-CD49a-CD103- |
| *osteoblast* | osteoblast |
|  | osteoclast |
| *pericyte* | pericyte cell |
| *plasma_cell* | plasma cell |
| *stem_cell* | GM23338 originated from GM23248 |
|  | GM23338 originated from GM23249 |
|  | GM23338 originated from GM23250 |
|  | GM23338 originated from GM23251 |
|  | H1-hESC |
|  | H1-hESC_stem cell |
|  | H7-hESC |
|  | hematopoietic multipotent progenitor cell |
|  | hematopoietic multipotent progenitor cell |
|  | induced pluripotent stem cell |
|  | mesenchymal stem cell of adipose |
|  | mesenchymal stem cell of the bone marrow |
|  | mesenchymal stem cell of Wharton's jelly |
| *t_CD4* | CD4 |
|  | CD4-positive, alpha-beta T cell |
|  | Conventional CD4+ T cell |
|  | Naïve (CD127+CD25-CD45RA+) |
|  | Naive CD4 T cells |
|  | Naïve CD4 T cells |
|  | Naive CD4+ T cell |
|  | Naive CD4+ T Cells |
|  | Terminal effector CD4 T cells |
|  | Th1 cells |
| *t_CD4_effector* | CD4+ T effector |
| *t_CD4_memory* | CD4 memory T cells |
| *t_CD4_memory_central* | CD4+ Central Memory (CD45RA lo; CD197 (CCR7) hi) |
|  | central memory CD4-positive, alpha-beta T cell |
|  | naive CD4 purified cells |
|  | TCM CD3+CD4+CCR7+CD45RA- T cells |
|  | Tcm CD4 cell |
|  | TCM CD4 purified cells |
| *t_CD4_memory_effector* | CD4+ Effector Memory (CD45RA lo; CD197 (CCR7) lo) |
|  | effector memory CD4-positive, alpha-beta T cell |
|  | Effector memory T lymphocytes CSF2- |
|  | Effector memory T lymphocytes CSF2+ |
|  | TEM CD3+CD4+CCR7-CD45RA- T cells |
|  | Tem CD4 cell |
|  | TEM CD4 purified cells |
| *t_CD4_naive* | CD4+ Naive (CD45RA hi; CD197 (CCR7) hi) |
|  | naive CD4 purified cells |
| *t_CD8* | CD8 |
|  | CD8 T cells |
|  | CD8+ T cells |
|  | CD8-positive, alpha-beta T cell |
|  | Naive CD8 T cells |
|  | Naive CD8+ T Cells |
|  | Tc_22 |
|  | Tc1 |
|  | Tc17 |
|  | Tc17+1 |
|  | Tc2 |
|  | Terminal effector CD8 T cells |
| *t_CD8_memory* | CD8 memory T cells |
| *t_CD8_memory_central* | central memory CD4-positive, alpha-beta T cell |
|  | Central memory CD8 T cell |
|  | Tcm CD8 cell |
|  | TCM PD1-TIGIT |
| *t_CD8_memory_effector* | Tem CD8 cell |
|  | Effector memory CD8 T cells |
|  | effector memory CD8-positive, alpha-beta T cell |
|  | TEM |
|  | Terminally differentiated effector memory CD8 T cells |
| *t_CD8_naive* | activated naive CD8-positive, alpha-beta T cell |
| *t_cell* | CD3+ T cells |
|  | CD45+CD3+ T cells |
| *mait* | MAIT cells |
| *t_gamma_delta* | Vd2 gd T cells |
|  | gamma delta CD1- |
|  | Non-Vd2 gd T cells |
| *t_helper* | Th1/Th17 cells |
|  | CD4-positive, alpha-beta T cell |
|  | Follicular helper T cells |
|  | Tfh1 CD4+ T cell |
|  | Tfh1-17 CD4+ T cell |
|  | Tfh17 CD4+ T cell |
|  | Tfh2 CD4+ T cell |
|  | Th1/17 (CD127+CD25-CD45RA-CXCR3+CCR6+) |
|  | Th17+1 |
|  | Th22 |
|  | Th22 (CD127+CD25-CD45RA-CXCR3-CCR6+CCR4+CCR10+) |
| *t_follicular_helper* | Follicular helper T cells |
| *t_helper_h1* | Th1 |
|  | Th1 (CD127+CD25-CD45RA-CXCR3+CCR6-) |
|  | Th1 CD4+ T cell |
|  | Th1 cells |
| *t_helper_h17* | Th17 |
|  | Th17 (CD127+CD25-CD45RA-CXCR3-CCR6+CCR4+CCR10-) |
|  | Th17 CD4+ T cell |
|  | Th17 cells |
| *t_helper_h2* | CD3+CD4+CD45RA-CCR7+CD25-CCR4+ T cells |
|  | Th2 |
|  | Th2 (CD127+CD25-CD45RA-CXCR3-CCR6-CCR4+) |
|  | Th2 CD4+ T cell |
|  | Th2 cells |
| *t_helper_naive* | Naive helper T cells |
| *t_reg* | Regulatory CD4+ T cell |
|  | regulatory T cell |
|  | T regulatory cells |
|  | Treg 1/17 (CD127-CD25+CD45RA-CXCR3+CCR6+) |
|  | Treg 22 (CD127-CD25+CD45RA-CXCR3-CCR6+CCR4+CCR10+) |
|  | Treg CD4+ T cell |
|  | Treg1 (CD127-CD25+CD45RA-CXCR3+CCR6-) |
|  | Treg17 (CD127-CD25+CD45RA-CXCR3-CCR6+CCR4+CCR10-) |
|  | Treg2 (CD127-CD25+CD45RA-CXCR3-CCR6-CCR4+) |
