# Supplementary figures and images for: cellsig plug-in enhances CIBERSORTx signature selection for multidataset transcriptomes with sparse multilevel modelling
Source: Bioinformatics. 2023 Nov 11;39(12):btad685. doi: 10.1093/bioinformatics/btad685 (PMC10692870; doi:10.1093/bioinformatics/btad685)

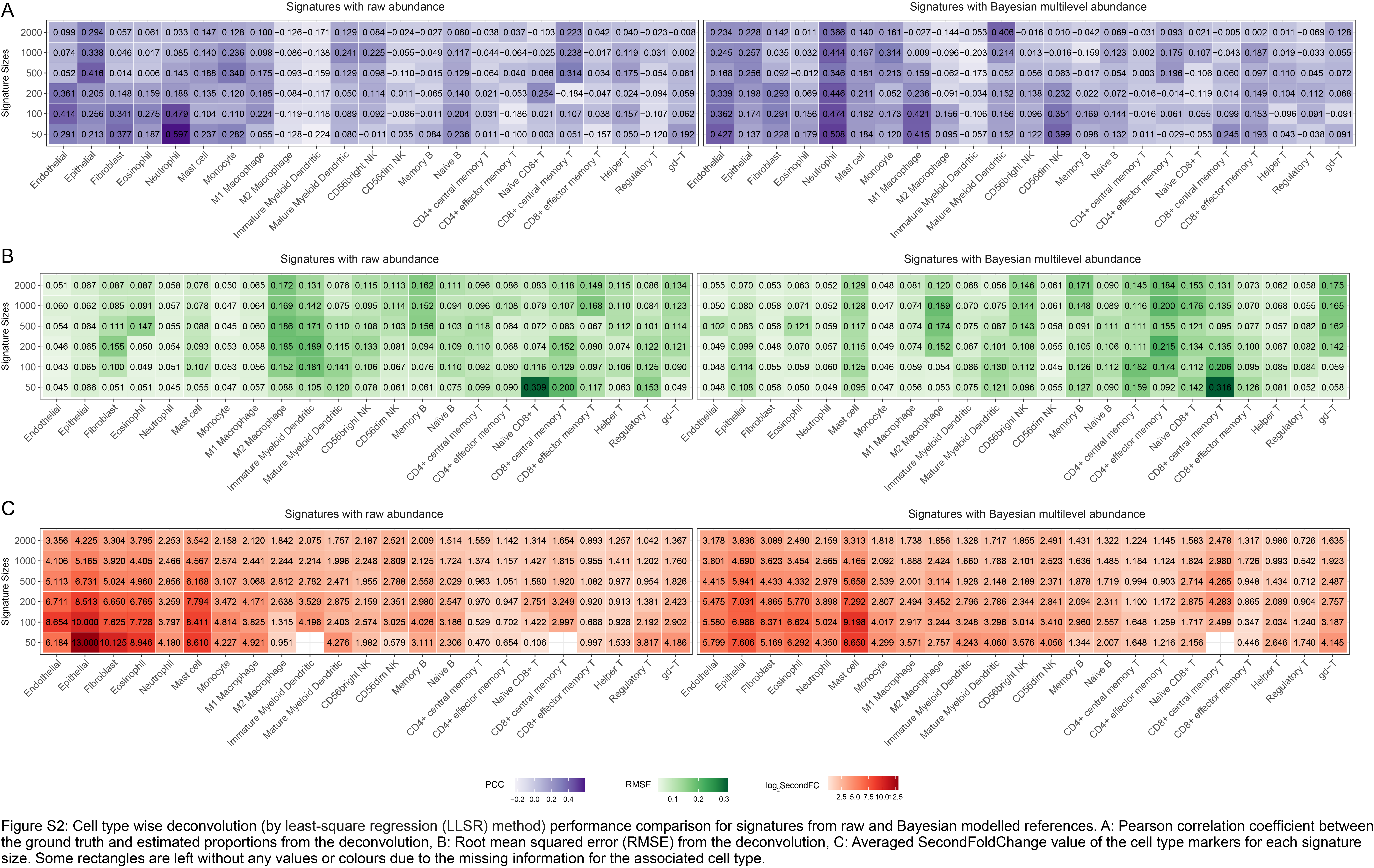

Supplement: btad685_Supplementary_Data [file btad685_supplementary_data.zip › Figure S2.png]

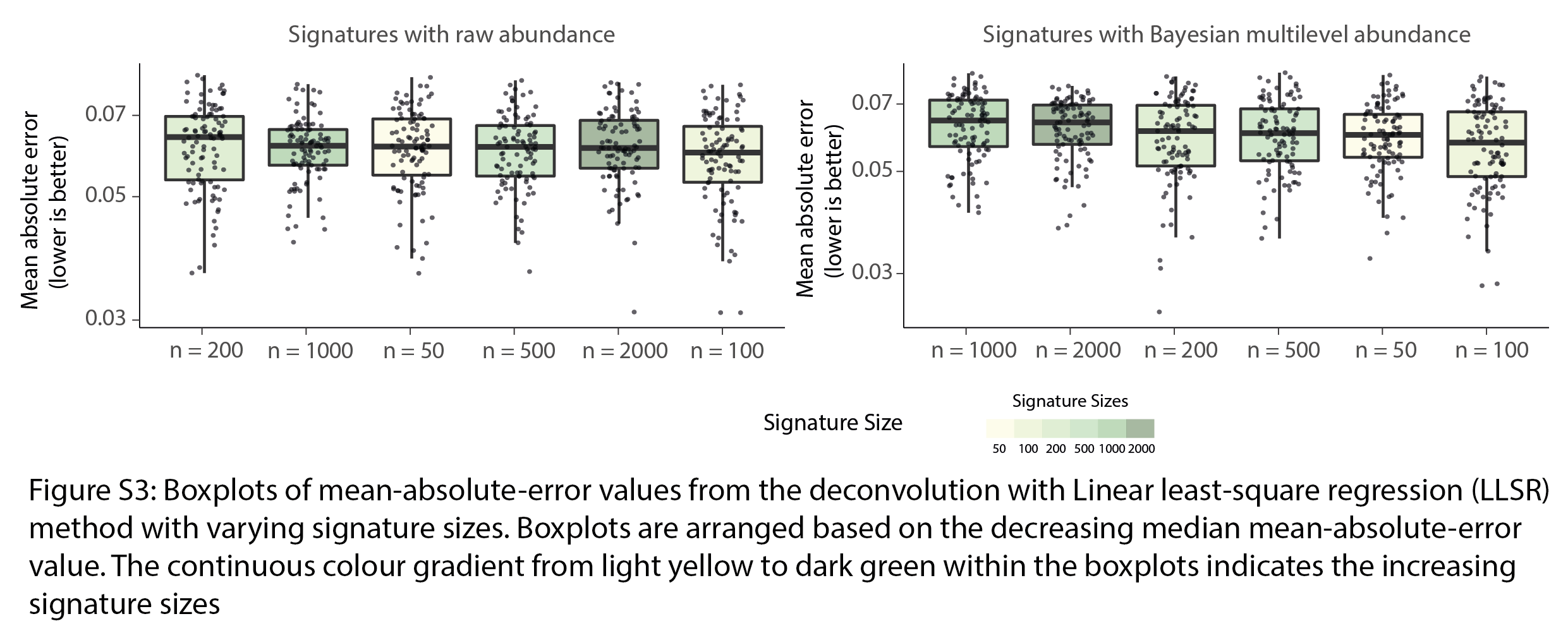

Supplement: btad685_Supplementary_Data [file btad685_supplementary_data.zip › Figure S3.png]

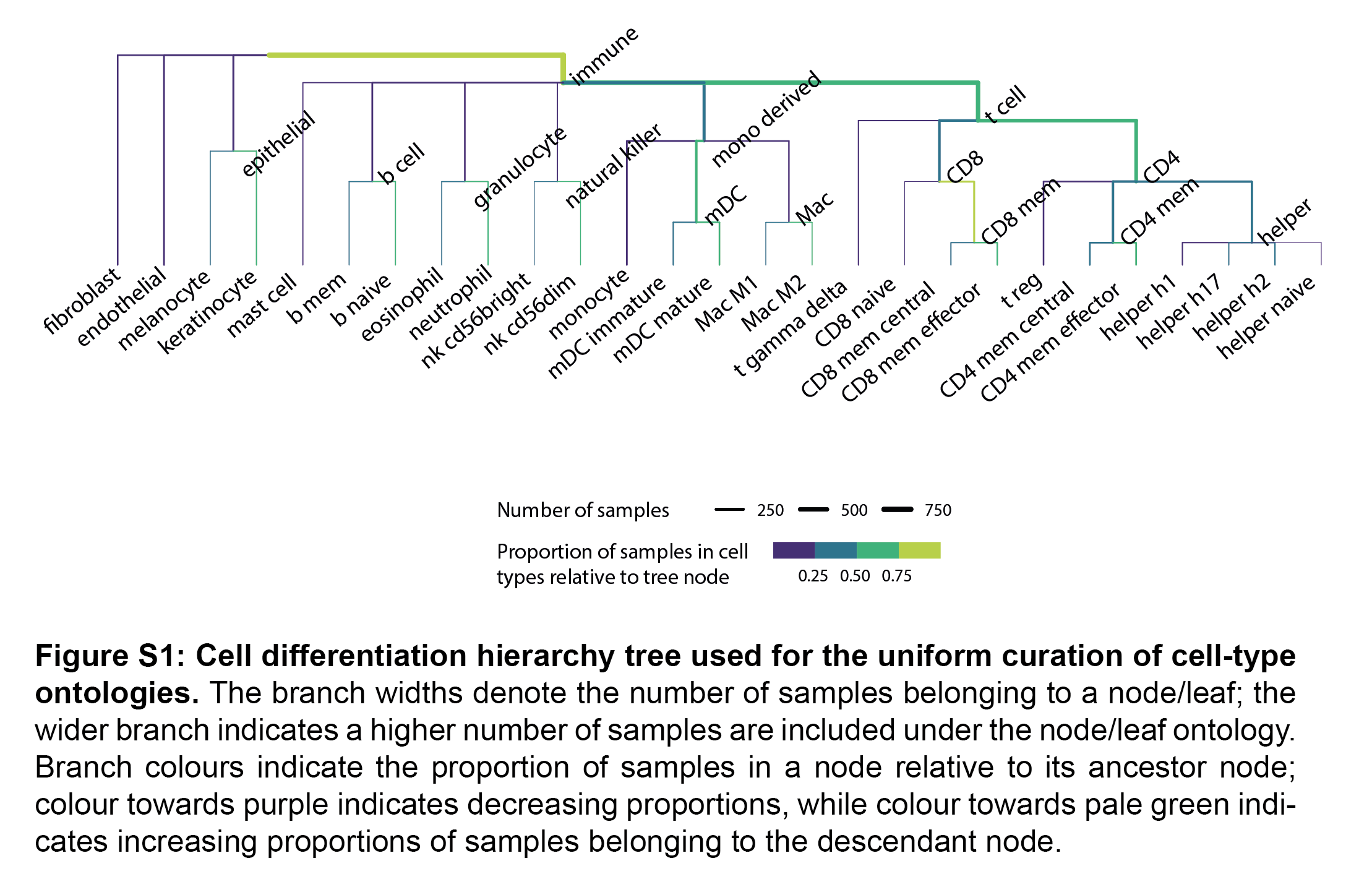

Supplement: btad685_Supplementary_Data [file btad685_supplementary_data.zip › Figure S1.png]
